# Cementless Tibial Fixation Results in Slower Recovery but Equivalent Outcome at 12 months in Primary Total Knee Arthroplasty

**DOI:** 10.1016/j.artd.2025.101792

**Published:** 2025-08-13

**Authors:** Khairul Anwar Ayob, Ishaan Jagota, Joshua Twiggs, David W. Liu

**Affiliations:** a The Gold Coast Centre for Bone and Joint Surgery, Palm Beach, Australia; b National Orthopaedic Centre of Excellence for Research and Learning, Department of Orthopaedic Surgery, Faculty of Medicine, University of Malaya, Kuala Lumpur, Malaysia; c Enovis ANZ, Sydney, Australia; d 360 Med Care, Sydney, Australia; e College of Science and Engineering, Flinders University, Adelaide, Australia

**Keywords:** Knee arthroplasty, Cementless, Cemented tibia

## Abstract

**Background:**

There is renewed interest in cementless primary total knee arthroplasty (TKA), driven by belief that robotics achieve more precise bone preparation and cementless fixation will improve survivorship, particularly in young patients. We undertook this study to investigate the early recovery trajectory of cementless tibial fixation using a contemporary design in primary TKA.

**Methods:**

One hundred and sixty-four propensity score-matched patients who underwent primary TKA with either cemented or reverse hybrid using cementless tibial components were retrospectively analyzed. Knee Injury and Osteoarthritis Outcome Score, Oxford Knee Score, Forgotten Joint Score-12, satisfaction and functional outcome measures including range of motion, sit to stand test, timed up and go, single leg stance, calf raises, and step count were compared between the 2 patient groups during the first 12 months postoperatively.

**Results:**

Patients with cemented tibial components had superior Knee Injury and Osteoarthritis Outcome Score scores at 3 and 6 months which was statistically significant. By 12 months, there was no difference in patient-reported outcome measures and overall satisfaction was similar. There was no difference between reverse hybrid and cemented tibial fixation in terms of range of motion and functional outcomes, mean length of stay, complication, blood transfusion, readmission, or reoperation. There were no failures or revisions in either group.

**Conclusions:**

Cementless tibial fixation resulted in slower improvement in patient-reported outcome measures during the first 3 to 6 months postoperatively. Patients planned for cementless tibial fixation should be counseled that their recovery may not be as rapid in the first 6 months as cemented fixation.

## Introduction

The number of total knee arthroplasties (TKAs) performed worldwide is increasing exponentially. Estimated future need for revision TKA is predicted to also expand and be proportionately higher [1,2]. Two cohorts of patients shown to have higher failure and revision rate include patients younger than 55 years and obese patients. In view of this, there is a need to develop and identify the ideal fixation method of the prosthesis to host bone to reduce the risk of implant loosening and the future burden of revisions.

Debate over the superiority of cemented and cementless fixation in TKA continues. Cement fixation has been regarded as the gold standard, with higher failure and revision rates involving previous cementless designs, especially for fixation of the tibia and patella [3]. Cementless fixation remains an attractive option however, given once osteointegration has been achieved the long-term results may be superior to cemented TKA. Cementless TKA may address the issues of radiolucency at the bone cement interface and subsequent loosening seen at longer term, particularly on the tibial side and in younger, higher-demand patients [4].

Despite having a good track record in TKA, cement does not “remodel” to accommodate higher stresses in the bone, and this could be associated with rates of loosening and debonding of cemented tibial components [5,6]. This issue is particularly pertinent to the obese patient. Improvements in porous metal fixation surface technology, and more precise and consistent bone preparation with robotic assistance may enhance the osseointegrative capabilities of cementless implants. Along with advances in the polyethylene wear properties, modern cementless TKA is an attractive option, particularly in young patients with good quality bone. However, evidence for the performance of contemporary cementless TKA is limited. Every cementless TKA design differs slightly in their macro and micro features and so each system needs to be assessed on its own merits and in individual studies. The tibial baseplate metallurgy and design has been postulated to induce stress shielding and lead to aseptic loosening of the prosthesis. This is thought to be more prevalent with tibial prostheses made from cobalt chromium (Co-Cr) [7,8]. However, analysis with quantitative dual-energy X-ray absorptiometry showed similar amounts of stress shielding between tibial trays of different thickness and material. Cementless prosthesis showed either similar or less stress shielding compared to their cemented equivalents. Additionally, surgeons commonly select cementless TKA in young active patients who require greater longevity. However, these patients are often still working and require an expedient recovery and return to function, to resume normal activities and capacity to earn income.

Some preliminary evidence from the Australian Orthopaedic Association National Joint Replacement Registry (AOANJRR) has indicated a higher-than-expected early failure rate with the Attune (J and J MedTech, Warsaw, Indiana) rotating platform cementless tibial base plate at 2 years [9]. In addition, it has been the senior authors anecdotal observation that patients with cementless TKA were painful for a longer period postoperatively. We therefore undertook this retrospective, propensity matched study to determine if there were any differences in the recovery path and early survivorship between the fully cemented and reverse hybrid TKA using a cementless tibial baseplate of the same design. Our hypothesis is the cemented version will have faster recovery, superior clinical outcome and lower failure rate than the cementless.

## Material and methods

Following ethics committee approval (Bellberry Human Research Ethics Committee, Sydney, Australia; No. 2012-03-710), we retrospectively evaluated 344 consecutive patients who underwent a primary TKA by the senior surgeon. Inclusion criteria comprised all patients between aged 45 and 90 in which primary TKA was performed using the Attune TKA prosthesis. Age, laterality, sex, comorbidities, American Society of Anaesthesiologists score, body mass index, length of stay (LOS), follow-up time, and indication for surgery were all retrieved from the electronic medical record. We excluded patients with missing perioperative data, and follow-up less than 2 years. Ninety-nine (28.7%) patients were excluded, of which 7 patients (2.0%) had missing perioperative data and 92 (26.7%) had not reached 2-year follow-up at the time of conducting the study. After exclusion of ineligible patients, 245 remained for analysis, which included 110 (44.9%) and 135 (55.1%) cemented and cementless tibial prosthesis respectively. Of the patients who met the inclusion criteria but were not matched (n = 81), 33.3% received a cemented prosthesis, while the rest (66.67%) received a cementless tibial prosthesis.

**Figure 1:**
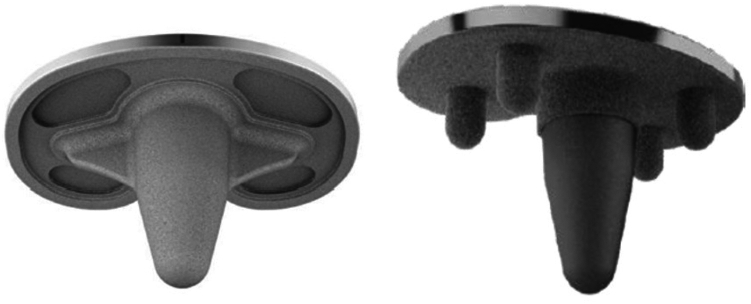
Cemented and cementless tibial base plates used in the study were identical apart from the undersurface fixation macro and micro characteristics.

The perioperative protocols were the same for all patients. Every patient received multimodal pain management, including periarticular infiltration of ropivacaine and tranexamic acid. The TKA was performed through a medial parapatellar approach without tourniquet and routine resurfacing of the patella. In the cemented group, 59 (71.95%) were performed with navigation (Brainlab Knee 3 Navigation, Munich, Germany), whereas 58 (70.73%) were conducted with navigation in the cementless cohort. The remainder of cases were performed with robotic assistance, using Velys Robotic-Assisted Solution (J and J Medtech, Warsaw, Indiana). All patients received the same rotating platform, posterior stabilized prosthesis (Attune, J and J Medtech, Warsaw, Indiana). In the cemented cohort, the femur, tibia and patella were fixed with Palacos R + G bone cement (Heraus, Hanau, Germany). The cementless cohort received an identical cemented femoral and patella component, with a press-fit tibial component made of cobalt chromium (Co-Cr) with sintered bead coating. Both tibial base plates were identical apart from the undersurface coating and macrofixation features (Fig. 1). Therefore, apart from the tibial component fixation, all other surgical variables were identical between the patient groups compared in the study.

During the initial period of patient inclusion all patients received the cemented tibial base plate due to unavailability of the cementless design. When the cementless tibial component was approved for use, the cementless design was utilized in all cases, except when based on the surgeon’s perception of tibial bone quality, it was deemed to be high risk of subsidence. After the time point that cementless implants became available, there were a subsequent 51 patients who received a cemented implant. The majority of patients received the cementless tibia once it was available.

The primary outcome was the Knee Injury and Osteoarthritis Outcome Score (KOOS), which was collected preoperatively, 3 months, 6 months, and 1 year postoperatively. Secondary outcome measures included the Oxford Knee Score (OKS), Forgotten Joint Score-12 and satisfaction scores. The satisfaction scores assessed patient satisfaction in terms of overall satisfaction, and satisfaction with pain and activities of daily living (ADLs). A visual analog scale was used to record the response, where a score of 0 indicated complete dissatisfaction and a score of 100 indicated complete satisfaction. Functional outcomes data included ROM, sit to stand test, timed up and go test, single leg stance test, and calf raises and was performed during the consultation session with the physiotherapist preoperatively and at 6 weeks postoperatively. Patient step counts from postoperative day 1 to day 42 was recorded using a wearable monitor (FitBit). LOS, complications, reoperations and revisions were also assessed for the study cohort.

### Data analyses

The sample size required was calculated a priori using G∗Power (Germany, version 3.1.9.6). Considering 5% marginal error, 80% power of study and medium effect size, 51 subjects were needed in each group to detect a difference in the mean outcome variables.

To control for selection bias between the groups, propensity score matching was performed to match the cemented to the cementless group. Patients were matched using a 1:1 ratio without replacement, employing a nearest neighbor logistic regression matching algorithm. The algorithm considered the following covariates: (1) age, (2) sex, (3) body mass index, (4) preoperative hip-knee angle, (5) fixed flexion deformity, (6) osteoarthritis grade, and (7) American Society of Anaesthesiologists score. The propensity score was defined as the conditional probability of receiving a cementless tibial prosthesis, with the outcome coded as 1 for cementless and 0 for cemented.

The study matched 82 knees in the cemented tibial group and 82 knees in the cementless tibial groups. Comparison of demographic parameters, disease severity and preoperative patient-reported outcome measures (PROMs) between the cohorts are outlined in Table 1. There were no significant differences in baseline characteristics as expected from the propensity matching.

**Table 1:** Demography and preoperative PROMs, (−) indicates varus deformity.

Study cohort characteristics and preoperative PROMs	Cumulative	Cemented	Cementless	*P* value
Demographics				
Gender (Men/Women)	(82/82)	(40/42)	(42/40)	.7565
Age (y)	69.83 ± 7.01	69.76 ± 6.46	69.9 ± 7.56	.8988
Body mass index (m/kg^2^)	29.67 ± 4.99	29.75 ± 5	29.59 ± 5.01	.8396
American Society of Anaesthesiologists	2.06	2.06	2.06	1
Disease severity				
Highest OA Grade	4.07 (±0.26)	4.06 (±0.24)	4.09 (±0.28)	.552
Preoperative HKA (deg)	−4.24 (±6.58)	−4.49 (±6.74)	−4 (±6.44)	.636
FFD (deg)	6.91 (±6.33)	6.76 (±6.49)	7.06 (±6.2)	.758
Preoperative PROMs				
KOOS Pain	49.36 (±14.05)	48.63 (±14.9)	50.09 (±13.2)	.514
KOOS symptoms	48.81 (±18.03)	47.83 (±18.44)	49.78 (±17.67)	.498
KOOS ADL	58.27 (±15.83)	57.51 (±15.33)	59 (±16.37)	.555
KOOS Sports	25.02 (±20.1)	24.07 (±20.1)	25.94 (±20.19)	.585
KOOS QOL	29.09 (±17.02)	28.96 (±18.3)	29.22 (±15.78)	.923
KOOS JR 100	51.75 (±10.62)	51.06 (±10.73)	52.42 (±10.53)	.425
SF-12 MCS	58.97 (±9.37)	59.14 (±8.41)	58.8 (±10.28)	.818
SF-12 PCS	31.33 (±7.56)	30.65 (±7.82)	32.00 (±7.28)	.263
OKS	26.42 (±6.71)	25.76 (±7.29)	26.81 (±6.38)	.52

CI, confidence interval; HKA, hip-knee angle; OKS, Oxford Knee Score; SF-12, 12-Item Short Form Survey; PCS, physical component summary (PCS) score; MCS, mental component summary (MCS) score; FJS, Forgotten Joint Score-12; OA, osteoarthritis.

*P* value <.05 considered to be statistically significant.

## Results

Statistically significant preoperative to postoperative improvements in PROMs were observed in the study population overall (table 2). Specifically, the KOOS pain at 12 months improved by 40.43 ± 16.71, symptoms by 34.78 ± 19.32, ADL by 33.69 ± 15.5, sports by 45.97 ± 22.51, quality of life (QOL) by 47.62 ± 24.30, Joint Replacement (JR) by 31.54 ± 14.42. SF-36 Physical Component summary (PCS) score and mental component summary (MCS) score also improved by 11.27 ± 8.79 and −1.13 ± 7.92 respectively.

**Table 2:** Postoperative PROMs overall and for the cemented and cementless subgroups at 3, 6 and 12 months postoperative.

Variable	Overall	Cemented	Cementless	*P* value	Mean difference	95% CI
Postoperative 3 mo						
KOOS JR	73.38 ± 10.76	76.33 ± 10.19	71.04 ± 10.74	.031	5.29	−1.671 to 5.451
KOOS						
Pain	78.1 ± 13.53	80.88 ± 11.76	75.84 ± 14.55	.095	5.04	−6.052 to 1.832
Symptoms	72.55 ± 15.55	76.29 ± 12.98	69.52 ± 16.9	.049	6.77	−1.907 to 7.427
ADL	84.77 ± 11.02	86.81 ± 10.19	83.11 ± 11.5	.136	3.7	−4.677 to 1.837
QOL	63.3 ± 17.77	69.82 ± 15.93	57.99 ± 17.59	.002	11.83	−4.179 to 7.659
MCS	57.68 ± 8.63	58.21 ± 7.58	57.26 ± 9.47	.625	0.95	−3.738 to 1.299
PCS	39.8 ± 6.51	40.86 ± 7.37	38.93 ± 5.66	.207	1.93	−3.202 to 1.002
Postoperative 6 mo						
KOOS-JR	76.11 ± 11.99	77.75 ± 10.98	74.44 ± 12.81	.147	3.31	−.360 to 6.980
KOOS						
Pain	83.61 ± 12.64	84.23 ± 11.47	82.99 ± 13.79	.606	1.24	−2.662 to 5.142
Symptoms	76.31 ± 14.92	79.08 ± 11.9	73.53 ± 17.08	.049	5.55	1.021 to 10.079
ADL	88.52 ± 10.06	88.81 ± 9.16	88.23 ± 10.97	.760	0.58	−2.529 to 3.689
QOL	69.64 ± 20.31	71.21 ± 18.45	68.08 ± 22.06	.418	3.13	−3.126 to 9.386
MCS	58.73 ± 8.62	58.03 ± 8.51	59.43 ± 8.75	.393	−1.4	−4.055 to 1.255
PCS	41.97 ± 6.7	41.99 ± 7.18	41.96 ± 6.25	.980	0.03	−2.041 to 2.101
Postoperative 12 mo						
KOOS JR	82.19 ± 12.91	83.16 ± 12.98	81.24 ± 12.88	.400	1.92	−2.058 to 5.898
KOOS						
Pain	88.63 ± 12.23	89.24 ± 11.85	88.05 ± 12.63	.582	1.19	−2.578 to 4.958
Symptoms	83.99 ± 13.04	85.94 ± 11.09	82.13 ± 14.51	.093	3.81	−0.163 to 7.783
ADL	91.39 ± 9.36	91.18 ± 9.88	91.59 ± 8.91	.802	−0.41	−3.304 to 2.484
QOL	76.91 ± 19.29	79.69 ± 17.11	74.25 ± 20.94	.106	5.44	−.0443 to 11.323
MCS	58.65 ± 8.72	58.31 ± 8.81	58.97 ± 8.69	.666	−0.66	−3.352 to 2.032
PCS	42.05 ± 6.74	42 ± 7.15	42.1 ± 6.37	.933	−0.1	−2.183 to 1.983
FJS-12	56.6667 ± 27.78	63.349 ± 26.463	54.531 ± 25.962	.299	7.338	−1.190 to 15.866

CI, confidence interval; SF-12, 12-Item Short Form Survey; PCS, physical component summary (PCS) score; MCS, mental component summary (MCS) score; FJS, Forgotten Joint Score-12.

Values are expressed as mean ± SD.

*P* value <.05 considered to be statistically significant.

### Comparison between cemented and cementless groups

**Figure 2:**
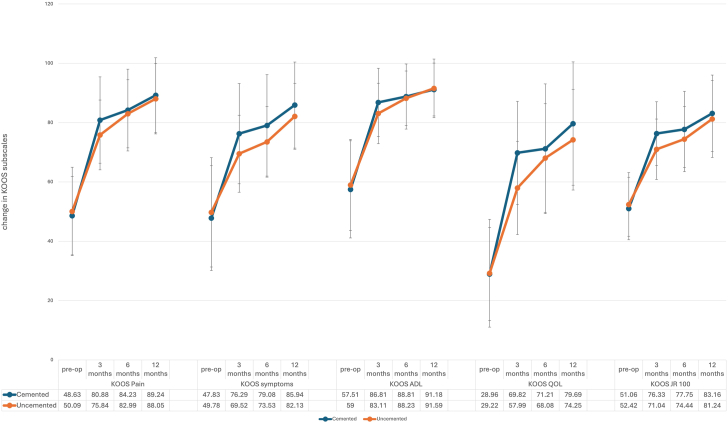
Comparison of change in KOOS subscores between cemented and cementless tibial cohorts at 3, 6 and 12 months postoperatively.

**Figure 3:**
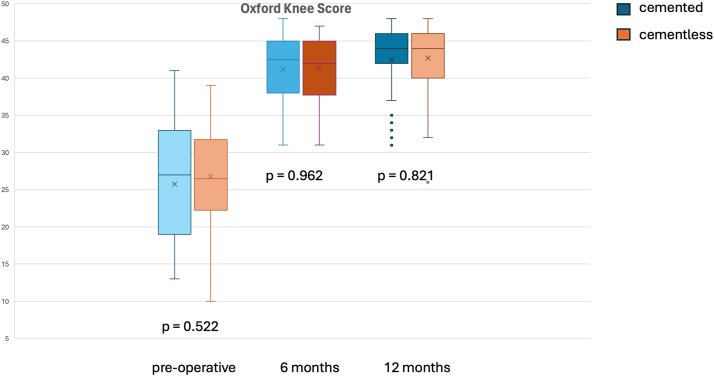
Boxplot showing Oxford Knee Scores preoperatively and at 6 and 12 months after surgery in cemented vs cementless tibial fixation.

The cemented group demonstrated a greater improvement in KOOS scores in the early postoperative period (Fig. 2 and table 3), with the cementless group drawing level at 12 months postoperatively. Specifically, the KOOS pain scores at 3 months improved by 36.04 in the cemented group, compared to 27.30 in the cementless group and this was statistically and clinically significant. Similarly, the early improvements in the KOOS symptoms, ADLs, QOL, and KOOS JR scores were better and statistically significant in the cemented group. However, using the minimal clinical important differences (MCID) determined from previous studies [10], only the differences in KOOS symptoms and QOL were clinically meaningful, while the KOOS pain, ADLs and KOOS JR were not [11,12]. There was progressive improvement with satisfaction scores, continuing to the 12-month mark. There was no significant difference in satisfaction between the cemented and cementless patient groups at any time point (table 4) or Oxford Knee Score (Fig. 3).

**Table 3:** Mean change in KOOS subscores at each follow-up time point for cemented and cementless patients.

Change in scores	Cumulative	Cemented	Cementless	*P* value
3 mo				
KOOS Pain	31.22	36.04	27.30	.024
KOOS symptoms	24.66	31.08	19.44	.022
KOOS ADL	28.29	33.45	24.20	.030
KOOS Sports	37.09	42.5	33.48	.221
KOOS QOL	35.74	43.21	29.65	.005
KOOS JR 100	22.84	27.73	19.09	.010
6 mo				
KOOS Pain	35.27	38.22	32.64	.094
KOOS symptoms	27.16	33.07	21.88	.008
KOOS ADL	30.06	32.98	27.51	.093
KOOS Sports	40.37	43.67	38.08	.338
KOOS QOL	39.27	42.5	36.38	.221
KOOS JR 100	25.15	29.45	21.32	.002
12 mo				
KOOS Pain	40.43	40.74	40.09	.847
KOOS symptoms	34.78	37.82	31.42	.099
KOOS ADL	33.69	33.50	33.91	.896
KOOS Sports	45.97	47.76	44.40	.526
KOOS QOL	47.62	50.25	44.70	.263
KOOS JR 100	31.5	32.91	30.04	.328

**Table 4:** Satisfaction scores at postoperative follow-up time points.

Satisfaction	3	6	12
Overall	83.93 ± 24.49	83.43 ± 26.95	92.38 ± 11.48
Cemented	86.95 ± 23.63	80.4 ± 31.48	93.15 ± 9.59
Cementless	81.17 ± 25.14	86.45 ± 21.12	91.61 ± 13.11
Pain	79.2 ± 31.66	83.43 ± 29.72	91.02 ± 19.71
Cemented	80.2 ± 33.5	80.91 ± 33.8	89.88 ± 22.66
Cementless	78.28 ± 30.13	86.04 ± 24.85	92.15 ± 16.38
ADL	83.58 ± 26.44	89.21 ± 20.12	92.06 ± 17.62
Cemented	84.44 ± 28.56	89.17 ± 22.66	91.98 ± 18.85
Cementless	82.8 ± 24.55	89.25 ± 17.3	92.13 ± 16.46

### Functional assessments

The functional outcomes preoperatively, 6 weeks postoperatively and change in functional outcomes are summarized in table 5. Preoperatively, the mean number of calf raises performed in the cementless group was significantly less and persisted at the reassessment period at 6 weeks postoperatively. Improvement in knee extension, as well as the 30 second sit to stand tests were also more pronounced in the cemented group. The average step count in the cemented group was less than that of the cementless group but was not statistically significant (table 6). Average LOS was 4.63 ± 1.71 days for the cemented patients compared to 4.53 ± 1.49 days for the cementless cohort with no significant difference.

**Table 5:** Preoperative and postoperative Functional Assessment Measurements for the cemented and cementless tibia patients.

Variable	Overall	Cemented	Cementless	*P*	Mean diff	95% CI
Preoperative						
Extension	5.29 ± 4.43	5.58 ± 4.39	5.01 ± 4.48	.431	.57	−0.794 to 1.934
Flexion	115.35 ± 18.62	115.04 ± 14.16	115.65 ± 22.25	.840	−0.61	−6.347 to 5.128
Sit to stand	9.05 ± 5.22	8.66 ± 4.93	9.43 ± 5.49	.362	−0.77	−2.375 to 0.835
Timed up and go	8.96 ± 3.58	9.13 ± 3.72	8.78 ± 3.44	.557	0.35	−0.752 to 1.452
Calf raise	14.09 ± 5.55	15.63 ± 6.49	12.54 ± 3.85	<.0001	3.09	1.448 to 4.732
6 wk						
Extension	2.07 ± 2.55	1.84 ± 2.44	2.33 ± 2.66	.333	−0.49	−1.275 to 0.295
Flexion	116.22 ± 9.71	115.71 ± 10.62	116.80 ± 8.63	.566	−1.09	−4.067 to 1.887
Sit to stand	10.72 ± 4.9	11.07 ± 4.04	10.33 ± 5.75	.450	0.74	−0.789 to 2.269
Timed up and go	7.40 ± 1.77	7.51 ± 1.94	7.27 ± 1.56	.496	0.24	−0.302 to 0.782
Calf raise	18.26 ± 6.8	20.91 ± 7.19	15.17 ± 4.77	<.0001	5.74	3.863 to 7.617
Change						
Extension	−2.74 ± 4.43	−3.92 ± 4.24	−1.45 ± 4.31	.00433	−2.47	−3.785 to −1.155
Flexion	0.56 ± 20.43	0.02 ± 12.04	1.14 ± 26.84	.789	−1.12	−7.519 to 5.280
Sit to stand	0.80 ± 4.84	1.94 ± 3.8	−0.43 ± 5.53	.0142	2.37	0.910 to 3.830
Timed up and go	−1.13 ± 1.79	−1.21 ± 1.55	−1.05 ± 2.05	.679	−0.16	−0.719 to 0.399
Calf raise	3.56 ± 5.4	4.58 ± 6.04	2.38 ± 4.33	.040	2.2	0.583 to 3.817

CI, confidence interval.

**Table 6:** Mean step counts during the first 6 weeks postoperatively, comparing the cemented and cementless groups.

Steps	Cemented		Cementless		*P* value
1 wk	1431.73 (±1091.12)	30 (36.59)	1905.03 (±1751.12)	30 (36.59)	0.215
2 wks	2633.78 (±1781.2)	37 (45.12)	3323.59 (±2485.91)	29 (35.37)	0.213
4 wks	4871.39 (±3362.96)	33 (40.24)	5837.18 (±3063.02)	22 (26.83)	0.276
6 wks	5100.88 (±3329.8)	8 (9.76)	5931.17 (±3733.6)	6 (7.32)	0.675

### Readmissions and complications

There was 1 readmission in the cemented group for persistent wound ooze. This resolved with rest and antibiotics, without the need for reoperation and the patient was discharged 7 days after readmission. Another patient in the cemented group developed a complex regional pain syndrome, necessitating referral to a pain specialist. The patient’s complex regional pain syndrome resolved by 12 months and she was satisfied with the result. There was no readmissions or complications reported in the cementless group.

### Revisions and reoperations

Four further procedures were required in the study cohort, with 1 patient in the cemented group and 2 patients in the cementless group requiring manipulation under anesthesia, performed between 4-8 months after surgery. All 3 patients subsequently reported a satisfactory result with no further intervention required following the manipulation under anesthesia. An arthroscopic debridement for patella clunk due to a peripatellar nodule was performed in the cemented tibia group at postoperative 8 months. There were no infections, component loosening or revisions in any patients included in the study up to 2 years postoperatively.

## Discussion

The main finding of our study was that the cementless tibial fixation cohort had a slower improvement in PROMs during the first 6 months before equalizing at 12 months postoperative. There was also greater improvement in knee extension and number of sit to stand repetitions in the cemented patients at 6 weeks. Both cementless and cemented tibial fixation had equivalent outcomes at 12 months postoperatively, with no difference in PROMs, satisfaction, revision or reoperation rate. The interest in cementless fixation for TKA has seen a resurgence, with the emergence of new materials for biological ingrowth, advances in manufacturing technology and popularity of robotics, believed to deliver a superior bone surface for cementless fixation [4,[13], [14], [15]]. Fully cementless fixation is an attractive option in patients traditionally at high risk for loosening such as the obese and young, and a way of improving operative efficiency. Our study is unable to draw any conclusions on operative efficiency as all patients received a cemented femur and patella, and this is unlikely to reduce operative time over fully cemented TKA. Our study highlights that patients with cementless tibial fixation may need to be warned that their recovery may not be as rapid as with cemented fixation, as a tradeoff for potentially greater implant longevity.

Historically, cementless designs have been associated with higher clinical failure rate, with the patella, and to a lesser extent, the tibial component being the traditional mode of failure [3,16]. These revisions were observed to occur in the 1.5-to-2-year period, after which, the rate of revision between the 2 methods of fixation equalizes [[17], [18], [19]]. The cementless tibial design used in the current study has been associated with a higher-than-expected revision rate at 2 years in the AOANJRR in 2024 [9] and this was partly the impetus to perform the current study. It is well known that cementless tibial fixation is sensitive to area of bone contact and degree of press-fit and therefore highly technique dependent [[20], [21], [22]]. In our study cohort, there were no fixation failures or revisions for loosening up to 2 years postoperative, so we believe we are unlikely to see the higher failure rate noted in the AOANJRR.

Our study finding of the initial improvement of symptoms being more pronounced in the cemented group was similar to Ko *et al.* [23] who analyzed PROMs from patients enrolled in the Comparative Effectiveness of Pulmonary Embolism Prevention after Hip and Knee Replacement trial. Although the main objective of this study was to determine safety of anticoagulants postarthroplasty, they reported KOOS JR scores at 6 months were 3.3 points greater in the cemented group. However, they were not able to standardize prosthesis type, bearing constraint, or surgical technique in their analysis. A randomized controlled study by Nam *et al.* [24] compared cementless and cemented tibial fixation using the same TKA implant brand as our study. They reported no difference in the change in PROMs between the groups at 6 weeks, 1 year and 2 years postsurgery. The tibial design in their study however was a 3D-printed baseplate with a highly porous titanium coating. The difference in porous coating and metallurgy may be responsible for the difference between Nam’s study and ours. A prospective randomized study by Fricka *et al.* [25] had similar early results to our study, with marginally inferior PROMs at the 2 year mark in cementless TKA. This study analyzed a trabecular metal pegged tibial tray with no keel, a different prosthesis design compared to the current study. Another difference in our study compared with previous literature was the use of a rotating platform, posterior-stabilized insert. The bearing is theoretically able to offset rotational forces on the tibial tray. This reduces the motion at the tibial tray–bone interface [26], which may increase the chances of successful osteointegration. Our short-term results however are consistent with those of a fixed-bearing prosthesis, which shows slower improvement in PROMs in the cementless cohort [23,25].

One of the pitfalls of cementless TKA is that the bone surfaces are not always prepared in a manner where contact between the prosthesis and bone is fully maximized. Conventional instrumentation utilizing a slotted cutting guide can result in deviation of the saw blade around sclerotic bone, leading to uneven bone cuts. This reduces the surface area for initial stability and osteointegration to occur. The recent introduction of robotic-assisted TKA has been proposed to lead to a more uniform cut surface for contact with the porous surface of cementless components [27,28]. A flat tibial cut is important for prosthesis stability and osteointegration. We found no differences in the overall short term outcome between different methods of instrumentation [29] in our study comparing computer navigation which required a slotted cutting block and a robotic arm guided saw, another potential strength of our study.

Although 40% ingrowth is considered to be adequate [30], modern cementless TKA designs have shown earlier and higher quality surface ingrowth [31]. Advancement in metallurgy, surface coating and fixation technology is rapidly changing and every cementless TKA varies slightly in features. Each design should be evaluated individually on their own merits, rather than grouping all cementless prostheses together and making generalizations. This study focuses on a specific implant design used in contemporary practice. We hypothesize there are minor differences in the primary stability of the tibial baseplate between cemented and cementless fixation in the initial period, which translated to slightly poorer PROMs in the cementless tibial group in the first 6 months postoperatively. However, with improvement in stability as osteointegration occurs, PROMs start to equalize by 12 months postoperatively. The minor “instability” was not significant enough to lead to failure of osseointegration at the prosthesis-bone junction, hence we did not observe any revisions or failures in the cementless cohort.

This study has several limitations. The surgeries were performed by a single surgeon from a single tertiary care center and may not be generalizable to all surgeons. A specific rotating platform design was used and the results may not represent different implant designs. We acknowledge that hybrid TKA, in which the femur is cemented and tibia cementless, is not a frequently utilized combination and has no proven benefits over all cemented or all cementless TKA. Additionally, we recognize that there would be little benefit in time saving and resources while cementing the femur and patella but not cementing the tibia. However, during the period from which the study patients were drawn, the cementless femur and patella were still awaiting regulatory clearance and could not be accessed. This scenario presented us with the opportunity to perform a matched cohort study with only 1 variable being the tibial fixation. Isolating tibial cementless fixation as a single variable is a study strength, particularly as the tibial component is often viewed as more susceptible to loosening and failure than the femur. Hence, our study does add further insight to the current literature on cementless TKA. The study was retrospective with a small number of patients, who were not randomized to prosthesis type. Patients who were osteoporotic were not excluded, with the use of a cemented or cementless baseplate based on the surgeon’s discretion. While this potentially introduces another confounding variable to the study, intuitively the poor bone quality patients who received a cemented tibia would be at greater risk of inferior fixation and loosening. Therefore this should negatively impact the cemented cohort. Our study findings were the opposite, with the cementless group having inferior early outcomes.

This is a propensity score matched study based on demographics, degree of deformity and grade of arthritis and hence can be viewed as a direct comparison between cemented and cementless fixation with the same TKA design, other factors being equal. We did not collect step count data preoperatively and therefore cannot make any meaningful conclusions regarding steps postoperatively between the 2 groups apart from an observed postoperative difference. The study findings should not be misconstrued that all patients are candidates for cementless fixation. The duration of patient follow-up was relatively short and longer term outcomes of these study patients may not mirror these short term results.

## Conclusions

Cementless tibial fixation using a posterior-stabilized rotating platform prosthesis and chrome cobalt tibial baseplate resulted in slower improvement in PROMs in the first 3 to 6 months postoperatively. Patients who receive cementless tibial fixation in primary TKA may need to be counseled that their recovery and return to function may not be as rapid in the first 6 months as cemented tibial fixation.

## Conflicts of interest

Khairul Anwar Ayob is on the Speakers bureau/paid presentations for Stryker – Exeter Hip Course and is a Board member/committee appointments for the Malaysian Society for Hip and Knee Surgeons, ASM 2024. Ishaan Jagota is a paid employee of Enovis ANZ, Mathys Orthopaedics (subsidiary of Enovis), 360 Med Care (subsidiary of Enovis) and receives research support from Enovis, 360 Med Care (subsidiary of Enovis). Joshua Twiggs receives royalties from Zimmer Biomet; Speakers bureau/paid presentations for Zimmer Biomet, Depuy, LifeHealthCare, Stryker; Paid employee for Enovis ANZ, Mathys Orthopaedics (subsidiary of Enovis), 360 MedCare (subsidiary of Enovis); Paid consultant for Zimmer Biomet, Depuy; Unpaid consultants for Formus Lab, AthroLase; owns stock in Arthrolase, Formus Labs, Naviswiss; receives research support from Zimmer Biomet, Depuy, Enovis, Allay Therapeutics, 360 MedCare (subsidiary of Enovis); Editorial Board at Knee Surgery and Related Research and Editorial Board at Arthroplasty; Secretary, Arthroplasty Society of Australia, Young Surgeons Committee Chair, Asia Pacific Arthroplasty Society, Vice Chair, Knee Arthroplasty Subspecialty Committee, SICOT, and Malaysian Society of Hip and Knee Surgeons. David W. Liu receives royalties from Zimmer Biomet; is on the Speakers bureau/paid presentations for Zimmer Biomet, Depuy, LifeHealthCare; is a paid consultant for Zimmer Biomet, Depuy; is an unpaid consultant for Formus Labs and Arthrolase; owns stock in Arthrolase, Formus Labs, Naviswiss; receives research support from Zimmer Biomet, Depuy, Enovis, Allay Therapeutics; editorial board member at Knee Surgery and Related Research and Arthroplasty; secretary at Arthroplasty Society of Australia, young surgeons' committee chair at Asia Pacific Arthroplasty Society, and vice chair at Knee Arthroplasty Subspecialty Committee.

For full disclosure statements refer to https://doi.org/10.1016/j.artd.2025.101792.

## CRediT authorship contribution statement

**Khairul Anwar Ayob:** Writing – review & editing, Writing – original draft, Formal analysis. **Ishaan Jagota:** Formal analysis, Data curation. **Joshua Twiggs:** Formal analysis, Data curation. **David W. Liu:** Writing – review & editing, Supervision, Methodology, Conceptualization.

## Figures and Tables

**Conflict of Interest Statement for Twiggs:**
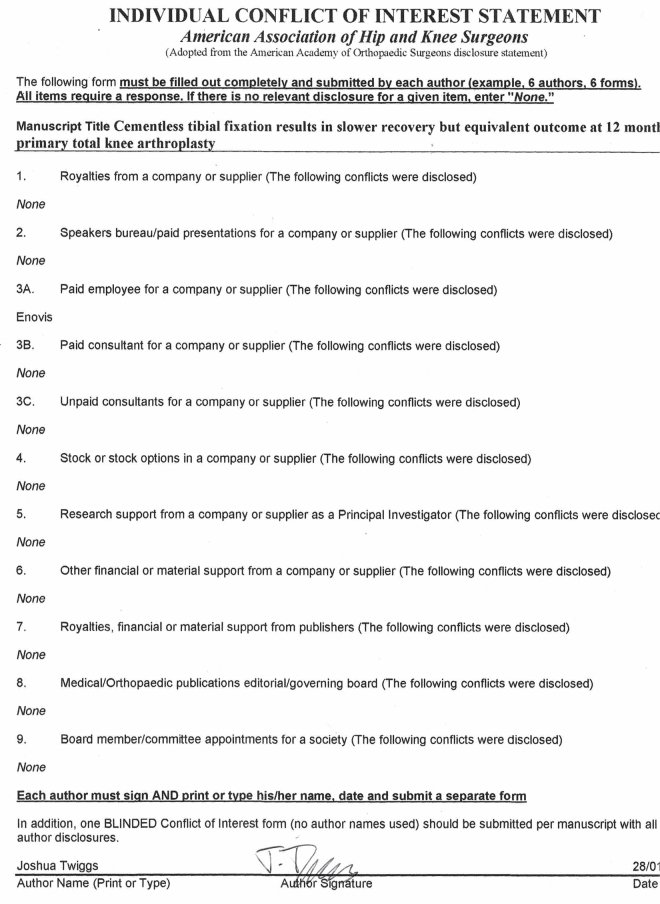


## Appendix A Supplementary data

Conflict of Interest Statement for Ayob

Conflict of Interest Statement for Jagota

Conflict of Interest Statement for Liu
