# Supplementary material for: Cementless Tibial Fixation Results in Slower Recovery but Equivalent Outcome at 12 months in Primary Total Knee Arthroplasty
Source: Arthroplast Today. 2025 Aug 13;35:101792. doi: 10.1016/j.artd.2025.101792 (PMC12362381; doi:10.1016/j.artd.2025.101792)
Supplement: Conflict of Interest Statement for Ayob [file mmc1.pdf]

INDIVIDUAL CONFLICT OF INTEREST STATEMENT

American Association of Hip and Knee Surgeons

(Adopted from the American Academy of Orthopaedic Surgeons disclosure statement)

The following form **must be filled out completely and submitted by each author (example, 6 authors, 6 forms). All items require a response. If there is no relevant disclosure for a given item, enter "*None.*"**

Manuscript Title Cementless tibial fixation results in slower recovery but equivalent outcome at 12 months in primary total knee arthroplasty

1. Royalties from a company or supplier (The following conflicts were disclosed)
Nil

2. Speakers bureau/paid presentations for a company or supplier (The following conflicts were disclosed)
Stryker – Exeter Hip Course

3A. Paid employee for a company or supplier (The following conflicts were disclosed)
Nil

3B. Paid consultant for a company or supplier (The following conflicts were disclosed)
Nil

3C. Unpaid consultants for a company or supplier (The following conflicts were disclosed)
Nil

4. Stock or stock options in a company or supplier (The following conflicts were disclosed)
Nil

5. Research support from a company or supplier as a Principal Investigator (The following conflicts were disclosed)
Nil

6. Other financial or material support from a company or supplier (The following conflicts were disclosed)
Nil

7. Royalties, financial or material support from publishers (The following conflicts were disclosed)
Nil

8. Medical/Orthopaedic publications editorial/governing board (The following conflicts were disclosed)
Nil

9. Board member/committee appointments for a society (The following conflicts were disclosed)
Malaysian Society for Hip and Knee Surgeons, ASM 2024

Each author must sign AND print or type his/her name, date and submit a separate form

In addition, one BLINDED Conflict of Interest form (no author names used) should be submitted per manuscript with all author disclosures.

Khairul Anwar, AYOB	[signature]	27th January 2025
Author Name (Print or Type)	Author Signature	Date
