# Supplementary material for: Cementless Tibial Fixation Results in Slower Recovery but Equivalent Outcome at 12 months in Primary Total Knee Arthroplasty
Source: Arthroplast Today. 2025 Aug 13;35:101792. doi: 10.1016/j.artd.2025.101792 (PMC12362381; doi:10.1016/j.artd.2025.101792)
Supplement: Conflict of Interest Statement for Jagota [file mmc2.pdf]

INDIVIDUAL CONFLICT OF INTEREST STATEMENT

American Association of Hip and Knee Surgeons

(Adopted from the American Academy of Orthopaedic Surgeons disclosure statement)

The following form **must be filled out completely and submitted by each author (example, 6 authors, 6 forms). All items require a response. If there is no relevant disclosure for a given item, enter "*None*."**

Manuscript Title **Cementless tibial fixation results in slower recovery but equivalent outcome at 12 months in primary total knee arthroplasty**

1. Royalties from a company or supplier (The following conflicts were disclosed)

   None

2. Speakers bureau/paid presentations for a company or supplier (The following conflicts were disclosed)

   None

3A. Paid employee for a company or supplier (The following conflicts were disclosed)
   Enovis ANZ, Mathys Orthopaedics (subsidiary of Enovis), 360 Med Care (subsidiary of Enovis)

3B. Paid consultant for a company or supplier (The following conflicts were disclosed)

   None

3C. Unpaid consultants for a company or supplier (The following conflicts were disclosed)

   None

4. Stock or stock options in a company or supplier (The following conflicts were disclosed)

   None

5. Research support from a company or supplier as a Principal Investigator (The following conflicts were disclosed)
   Enovis, 360 Med Care (subsidiary of Enovis)

6. Other financial or material support from a company or supplier (The following conflicts were disclosed)

   None

7. Royalties, financial or material support from publishers (The following conflicts were disclosed)

   None

8. Medical/Orthopaedic publications editorial/governing board (The following conflicts were disclosed)

   None

9. Board member/committee appointments for a society (The following conflicts were disclosed)
   None

Each author must sign AND print or type his/her name, date and submit a separate form

In addition, one BLINDED Conflict of Interest form (no author names used) should be submitted per manuscript with all author disclosures.

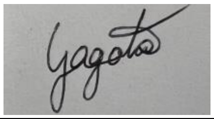

Ishaan Jagota		28/01/2025
Author Name (Print or Type)	Author Signature	Date
