# Supplementary material for: Cementless Tibial Fixation Results in Slower Recovery but Equivalent Outcome at 12 months in Primary Total Knee Arthroplasty
Source: Arthroplast Today. 2025 Aug 13;35:101792. doi: 10.1016/j.artd.2025.101792 (PMC12362381; doi:10.1016/j.artd.2025.101792)
Supplement: Conflict of Interest Statement for Liu [file mmc3.pdf]

INDIVIDUAL CONFLICT OF INTEREST STATEMENT

American Association of Hip and Knee Surgeons

(Adopted from the American Academy of Orthopaedic Surgeons disclosure statement)

The following form **must be filled out completely and submitted by each author (example, 6 authors, 6 forms). All items require a response. If there is no relevant disclosure for a given item, enter "*None.*"**

Manuscript Title: Cementless tibial fixation results in slower recovery but equivalent outcome at 12 months in primary total knee arthroplasty

1. Royalties from a company or supplier (The following conflicts were disclosed)

   Zimmer Biomet

2. Speakers bureau/paid presentations for a company or supplier (The following conflicts were disclosed)

   Zimmer Biomet, Depuy, LifeHealthCare

3A. Paid employee for a company or supplier (The following conflicts were disclosed)
   None

3B. Paid consultant for a company or supplier (The following conflicts were disclosed)
   Zimmer Biomet, Depuy

3C. Unpaid consultants for a company or supplier (The following conflicts were disclosed)
   Formus Labs, ArthroLase

4. Stock or stock options in a company or supplier (The following conflicts were disclosed)
   Naviswiss, Formus Labs Ltd, ArthroLase Pty Ltd

5. Research support from a company or supplier as a Principal Investigator (The following conflicts were disclosed)
   Zimmer Biomet, Depuy, Enovis, Allay Therapeutics

6. Other financial or material support from a company or supplier (The following conflicts were disclosed)
   None

7. Royalties, financial or material support from publishers (The following conflicts were disclosed)
   None

8. Medical/Orthopaedic publications editorial/governing board (The following conflicts were disclosed)
   Editorial Board, Knee Surgery and Related Research
   Editorial Board, Arthroplasty

9. Board member/committee appointments for a society (The following conflicts were disclosed)
   Secretary, Arthroplasty Society of Australia
   Young Surgeons Committee Chair, Asia Pacific Arthroplasty Society
   Vice Chair, Knee Arthroplasty Subspecialty Committee, SICOT

Each author must sign AND print or type his/her name, date and submit a separate form

In addition, one BLINDED Conflict of Interest form (no author names used) should be submitted per manuscript with all author disclosures.

David Liu	[signature]	20th February 2025
Author Name (Print or Type)	Author Signature	Date

Scanned with CamScanner
